# Efficacy and safety of Xuefu Zhuyu decoction combined with Western medicine for angina pectoris in coronary heart disease: A protocol for systematic review and meta-analysis

**DOI:** 10.1097/MD.0000000000023195

**Published:** 2020-12-11

**Authors:** Duode Wang, Ping Wang, Rong Zhang, Xiaoping Xi

**Affiliations:** Wuwei Hospital of Traditional Chinese Medicine, Wuwei, Gansu Province, China.

**Keywords:** Xuefu Zhuyu decoction, coronary heart disease, angina pectoris, protocol, systematic review

## Abstract

**Background:**

Angina pectoris in coronary heart disease (CHD) is a common ischemic heart disease clinically. During the onset, patients often have symptoms such as chest discomfort or paroxysmal crushing pain in the posterior sternum, which seriously affects the quality of life of patients, and even can lead to myocardial infarction and endanger the lives of patients. Clinical studies have shown that the compound Chinese prescription Xuefu Zhuyu decoction combined with western medicine has a certain therapeutic effect on angina pectoris in CHD, but lack of evidence of evidence-based medicine. The purpose of this study is to evaluate the efficacy and safety of Xuefu Zhuyu decoction combined with western medicine in the treatment of angina pectoris in CHD.

**Methods:**

Use computer to retrieve English databases (PubMed, Embase, Web of Science, the Cochrane Library) and Chinese databases (CNKI, Wan Fang, VIP, Chinese biomedical database), from the establishment of database to October 2020, for randomized controlled trials (RCTs) of Xuefu Zhuyu decoction combined with Western medicine for angina pectoris in CHD. Two investigators independently conducted data extraction and assessed the literature quality of the included studies. The Revman5.3 software was used for meta-analysis of the included literatures.

**Results:**

The efficacy and safety of Xuefu Zhuyu decoction combined with western medicine in the treatment of angina pectoris in CHD were evaluated by total effective rate, angina pectoris pain score, TCM syndrome score, electrocardiogram effect, hemorheology index (including whole blood viscosity, plasma viscosity, hematocrit, and fibrinogen), and the incidence of adverse reactions.

**Conclusion:**

This study will provide reliable evidence-based evidence for the clinical application of Xuefu Zhuyu decoction combined with western medicine in the treatment of angina pectoris in CHD.

**Ethics and dissemination:**

Private information from individuals will not be published. This systematic review also does not involve endangering participant rights. Ethical approval was not required. The results may be published in a peer-reviewed journal or disseminated at relevant conferences.

**OSF Registration number:**

DOI 10.17605 / OSF.IO / GFEQ7.

## 1 Introduction

Angina pectoris in coronary heart disease (CHD) is a syndrome caused by acute myocardial ischemia and hypoxia due to insufficient coronary blood supply.^[1]^ During the attack, patients often present with retrosternal crushing pain, which radiates to the shoulder and back, accompanied by fever, sweating, panic, nausea, etc, and the symptoms often last for several minutes.^[2,3]^ The etiology is often related to the narrowing of the inner diameter of the coronary artery lumen and the obstruction of blood circulation to some extent.^[4]^ Research shows that angina pectoris in CHD tend to occur in the middle-aged and elderly people. In recent years, with the change of lifestyle, the onset age of angina tends to be younger and poses a great threat to the life and health of modern people.^[5]^

According to Traditional Chinese medicine, angina pectoris in CHD belongs to the category of “xiong bi” (obstruction of qi in the chest) in Traditional Chinese medicine, and the key pathogenesis is blood stasis.^[6]^ Xuefu Zhuyu decoction originates from the Qing Dynasty doctor Wang Qingren's book *“Yilin Gaicuo*,*”* this prescription has the function of promoting blood circulation and removing blood stasis, and promoting qi circulation to relieve pain, which is the representative prescription for the treatment of xiong bi. According to the principle of treating different diseases with same method in Traditional Chinese medicine, after more than 100 years of clinical application, this prescription has a good effect on coronary atherosclerosis, hypertension, myocardial fibrosis, hyperlipidemia, angina, and other diseases.^[7,8]^

Clinical application of Xuefu Zhuyu decoction combined with western medicine in the treatment of angina pectoris in CHD has improved the clinical symptoms of patients and is more effective than that of western medicine alone.^[9]^ The results of several randomized controlled experiments in recent years show that Xuefu Zhuyu decoction combined with western medicine has the advantages of relieving angina pectoris symptoms, improving clinical treatment effect and low incidence of adverse reactions.^[10–12]^ However, the small sample size, the difference between the research scheme and the evaluation of curative effect make the research results uneven, which to some extent affect the reliability of the research results and the popularization of the clinical therapy. Based on the meta-analysis of Xuefu Zhuyu decoction combined with western medicine for angina pectoris in CHD, this article provides a reliable evidence-based basis for the wide application and scientific research of Xuefu Zhuyu decoction.

## 2 Methods

### 2.1 Protocol register

This protocol of systematic review and meta-analysis has been drafted under the guidance of the preferred reporting items for systematic reviews and meta-analysis protocols (PRISMA-P). What's more, it has been registered on the open science framework (OSF) on October 12, 2020. (Registration number: DOI 10.17605 / OSF.IO / GFEQ7.)

### 2.2 Ethics

Since this is a protocol with no patient recruitment and personal information collection, the approval of the ethics committee is not required.

### 2.3 Eligibility criteria

#### 2.3.1 Types of studies

We will collect all available randomized controlled trials (RCTS) on Xuefu Zhuyu decoction for the treatment of angina pectoris in CHD, regardless of blinding, publication status, or region, but the language is limited to Chinese and English.

#### 2.3.2 Research subjects

Patients diagnosed as angina pectoris in CHD according to the diagnostic criteria of CHD, excluding patients with other severe heart, liver and kidney dysfunction, and drug allergy. However, the patient's nationality, race, age, sex, course of disease are unlimited.

#### 2.3.3 Interventions

The control group: use western medicine treatment alone, the type, dosage, course of treatment is not limited. The treatment group: use Xuefu Zhuyu decoction on the basis of which in the control group, the dosage form, dosage, addition and subtraction of Xuefu Zhuyu decoction is not limited.

#### 2.3.4 Outcome indicators

1. Main indicators: total efficiency, the clinical efficacy evaluation was based on the *guidelines for clinical research of new Chinese medicines* issued by the Ministry of Health in 2002. Significant effect: After treatment, angina pectoris and other symptoms disappeared, and laboratory indicators showed normal; Effective: After treatment, the frequency of angina attack was significantly reduced, other symptoms were significantly improved, and laboratory indicators showed significant improvement; Invalid: not up to the above standard. Total effective rate = (number of significant cases + number of effective cases)/total number of cases 100%.
2. Secondary indicators:
  (a) TCM syndrome score: all symptoms were rated as 0 to 3 points according to their severity, 0 as none, 1 as mild, 2 as moderate, and 3 as severe^[13]^;
  (b) ECG;
  (c) Hemorheological parameters (including whole blood viscosity, plasma viscosity, hematocrit, and fibrinogen);
  (d) Incidence of adverse reactions.

### 2.4 Exclusion criteria

1. For the paper published repeatedly, take the most complete one;
2. Literatures whose data were incomplete or cannot be obtained after contacting the author;
3. Literatures with no relevant outcome indicators;
4. Literatures with obvious data errors;
5. Literatures with the control group and the treatment group included other treatments such as acupuncture and moxibustion, surgery, etc.

### 2.5 Retrieval strategy

Through the computer, the key words such as “xin jiao tong” (angina pectoris), “guan xin bing” (coronary heart disease), “xiong bi” (obstruction of qi in the chest), “zhen xin tong” (angina pectoris), “ Xuefu Zhuyu” were searched in Chinese databases such as CNKI, Wanfang, Weipu, China Biomedical Database (CBM), and so on; take “stenocardia,” “angina pectoris,” “coronary heart disease angina,” “Xuefu Zhuyu,” and so on as the English retrieval keywords to be retrieved in the PubMed, EMBASE, Web of Science, the Cochrane Library, and other English databases. In addition, manual retrieval is carried out on baidu academic, Google academic, books, impurities, and conference materials, so as to obtain the materials related to this study as comprehensively as possible. Take PubMed as an example, and the retrieval strategy is shown in Table 1.

**Table 1:** Search strategy in PubMed database.

Number	Search terms
#1	Angina pectoris [Mesh]
#2	Stenocardia [Title/Abstract]
#3	Angor pectoris [Title/Abstract]
#4	Coronary disease [Mesh]
#5	Coronary artery disease [Title/Abstract]
#6	Coronary heart disease angina [Title/Abstract]
#7	Angina, Stable [Mesh]
#8	Chronic stable angina [Title/Abstract]
#9	Stable angina pectori [Title/Abstract]
#10	#1 OR #2 OR #3 OR #4 OR #5 OR #6 OR #7 OR #8 OR #9
#11	Xuefu Zhuyu [Title/Abstract]
#12	Circulation Plus Decoction [Title/Abstract]
#13	#11 OR #12
#14	#10 AND #13

### 2.6 Data screening and extraction

According to the PRISMA flow chart, two researchers independently screened and retrieved the literature according to the inclusion and exclusion criteria, extracted relevant data, and cross-checked the methods of inclusion and exclusion according to the Cochrane Collaboration Handbook version 5.0. For studies with differences, the third researcher was consulted and resolved. First read the title and abstract of the article for screening, remove the literature that does not meet the inclusion criteria, and further read the full text for screening. At the same time, Excel2013 software is used to extract relevant information. Data extraction includes:

1. The first author's name, publication years;
2. Basic information of subjects: sample size, sex, age, and course of disease;
3. Interventions: Types of western medicine, dosage forms of Xuefu Zhuyu, composition, and course of treatment;
4. Outcome indicators and specific adverse reactions.

**Figure 1:**
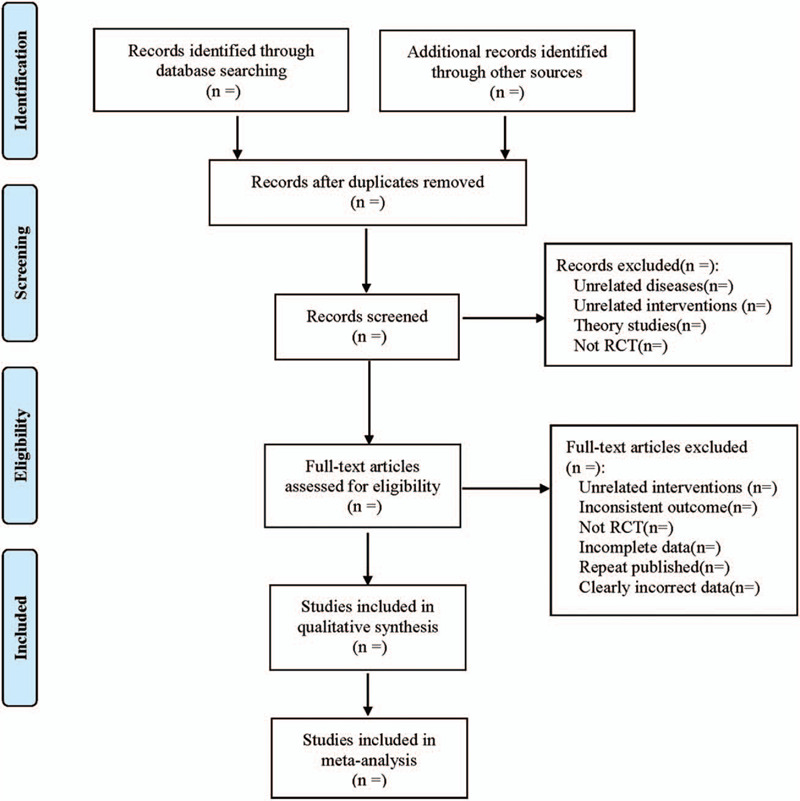
Flow diagram.

The literature screening process is shown in Figure 1.

### 2.7 Literature quality assessment

Risk Bias assessment was carried out for the included studies using the Risk of Bias assessment tool recommended by the Cochrane Reviewers Handbook 5.1.0. According to the performance of Random sequence generation, Aliocation concealment, Blinding of participants and personal, Blinding of outcome assessment, Incomplete outcome data, Selective reporting, Other bias and other aspects, the two researchers independently gave low risk, unclear and high risk judgment item by item, and carried out cross-checking after completion respectively. If there was any difference, they agreed with the third researcher.

### 2.8 Statistical analysis

The RevMan5.3 software provided by Cochrane collaboration network will be used to meta-analyze the extracted data. For continuous variables, if the unit or measuring tool of the measurement index is consistent, the mean difference (MD) is used as the statistic, and if not, the standard mean difference (SMD) is used as the statistic. The relative risk degree (RR) is used as the effect analysis statistic, the *χ*^*2*^ test is used for the heterogeneity among the included studies, and *I*^2^ is used to quantitatively judge the heterogeneity between studies. If (*P* ≥ .1, *I*^2^ ≤ 50%), the heterogeneity among studies is low, and the fixed-effect model is used for Meta analysis; if (*P* < .1, *I*^2^ > 50%), it is indicated that there is significant heterogeneity the studies, and the source of heterogeneity should be further analyzed. If there is obvious clinical heterogeneity, subgroup analysis is carried out; if clinical heterogeneity is obvious and subgroup analysis cannot be carried out, meta-analysis is not carried out, only descriptive analysis is used; if there is no obvious clinical and methodological heterogeneity, statistical heterogeneity should be considered and random effect model is used for meta-analysis.

#### 2.8.1 Dealing with missing data

If there is a lack of data in the article, contact the author to supplement the missing information. If the author cannot be contacted, or the author has lost the relevant experimental data, the meta-analysis is not carried out, but descriptive analysis is used.

#### 2.8.2 Subgroup analysis

Subgroup analysis was carried out according to the types of combined western medicine; subgroup analysis was carried out according to different types of angina pectoris; subgroup analysis was carried out according to the course of treatment; subgroup analysis was carried out according to the dosage form of Xuefu Zhuyu decoction.

#### 2.8.3 Sensitivity analysis

Sensitivity analysis was used to observe the effect of single outcome index on the amount of combined effect to judge the stability of meta-analysis results.

#### 2.8.4 Assessment of reporting biases

When the number of main indicators in the studies included was more than 10, the funnel plot was used to evaluate the publication bias. Moreover, Egger's and Begg's test were used for the evaluation of potential publication bias.

## 3 Discussion

Angina pectoris in CHD belongs to the category of “xiong bi” (obstruction of qi in the chest), “zhen xin tong” (angina pectoris) in Traditional Chinese medicine.^[14]^ In recent years, the incidence of CHD and angina pectoris has shown significant characteristics. During the clinical attack, patients not only have pain in the anterior cardiac region, but also their psychological state and quality of life have been seriously affected.^[15]^ At present, modern clinical use nitroglycerin, β-receptor blockers, calcium channel blockers and other western medicine to treat angina pectoris in CHD. However, long-term medication may cause side effects such as gastrointestinal bleeding, decreased heart rate or blood pressure, and the long-term efficacy is not satisfactory.^[16]^ Therefore, it is necessary to find a treatment for angina pectoris with fewer side effects and more significant curative effect.

Traditional Chinese medicine believes that the emergence of “xiong bi” is mostly caused by deficiency of qi and blood stasis blocking the choroid, and its pathogenesis is mainly due to deficiency of qi. Deficiency is mainly due to the deficiency of qi, blood, yin, and yang in the body, including phlegm obstruction, cold coagulation, qi stagnation, and blood stasis, that is, "if dredge, there will not pain, pain is because of impassability. Xuefu Zhuyu decoction, as a classical prescription of traditional Chinese medicine with the function of activating blood circulation and removing blood stasis, can remove blood stasis, carry out stagnation of qi, and effectively alleviate the clinical symptoms of patients.^[17,18]^ The prescription consists of 11 herbs: Taoren (*Semen Persicae*), Honghua (*Flos Carthami*), Danggui (*Radix Angelicae Sinensis*), Shengdi (*Radix Rehmanniae*), Chuanxiong (*Rhizoma Chuanxiong*), Chishao (*Radix Paeoniae Rubra*), Niuxi (*Radix Cyathulae*), Jiegeng (*Radix Platycodonis*), Chaihu (*Radix Bupleuri*), Zhiqiao (*Fructus Aurantii*), Gancao (*Radix Glycyrrhizae*). In the prescription, Taoren (*Semen Persicae*), Honghua (*Flos Carthami*), Danggui (*Radix Angelicae Sinensis*), Chuanxiong (*Rhizoma Chuanxiong*), Chishao (*Radix Paeoniae Rubra*) have the function of activating blood circulation and removing blood stasis; Shengdi (*Radix Rehmanniae*) nourish yin and nourish blood, and promote blood circulation combined with Danggui (*Radix Angelicae Sinensis*); Chaihu (*Radix Bupleuri*) disperse stagnated liver qi for relieving qi stagnation; Jiegeng (*Radix Platycodonis*) and Zhiqiao (*Fructus Aurantii*), one lift, one drop, regulate qi activity; Niuxi (*Radix Cyathulae*) promote blood circulation and remove blood stasis, ensure proper downward flow of the blood; Gancao (*Radix Glycyrrhizae*) coordinate the drug actions of a prescription. All drugs combined together, promote and demote qi, harmonize qi and blood, playing the function of removing blood stasis, promoting qi, and relieving pain.

Modern clinical studies have found that the pathogenesis of angina pectoris in CHD is mainly due to atherosclerosis, or plaque formation in the inner wall of blood vessels, leading to stenosis of the lumen, or changes in hemorheology. For example, increased blood viscosity leads to slow blood flow affecting myocardial blood supply.^[19,20]^ Experimental studies have shown that Xuefu Zhuyu decoction can resist myocardial cell apoptosis and has the biological activity of protecting the heart. It can significantly reduce the viscosity of whole blood and plasma, significantly prolong the clotting time, inhibit platelet aggregation, increase coronary blood flow, improve cardiac microcirculation, and reduce plaque formation.^[21–23]^ At present, although some research results have confirmed that Xuefu Zhuyu decoction combined with Western medicine can significantly alleviate the condition of patients with angina pectoris in CHD, its clinical efficacy has not been recognized by international authoritative medical organizations.^[24]^ Therefore, it is necessary to carry out meta-analysis on the results of the existing randomized controlled studies on the treatment of CHD angina with Xuefu Zhuyu decoction combined with western medicine, so as to provide reliable basis for the clinical application of Xuefu Zhuyu decoction in the treatment of CHD angina. However, this study also has some limitations. Due to the low quality of the original study included and the limited sample size included, the accuracy of the research results is affected to some extent. At the same time, due to the limitation of language ability, our retrieval scope was only limited to Chinese and English literature, and we may ignore the studies or reports in other languages. Due to the particularity of TCM decoction, the composition and dosage of Xuefu Zhuyu decoction used in each study were different to some extent, resulting in certain heterogeneity of research results.

## Author contributions

**Funding acquisition:** Ping Wang.

**Investigation:** Xiaoping Xi.

**Project administration:** Duode Wang.

**Software:** Xiaoping Xi.

**Supervision:** Rong Zhang.

**Writing – original draft:** Duode Wang, Rong Zhang.

**Writing – review & editing:** Ping Wang.
